# Effect of Domain Structure of Segmented Poly(urethane-imide) Membranes with Polycaprolactone Soft Blocks on Dehydration of *n*-Propanol via Pervaporation

**DOI:** 10.3390/polym10111222

**Published:** 2018-11-03

**Authors:** Maria P. Sokolova, Alexander N. Bugrov, Michael A. Smirnov, Alexander V. Smirnov, Erkki Lahderanta, Valentin M. Svetlichnyi, Alexander M. Toikka

**Affiliations:** 1 Department of Chemical Thermodynamics & Kinetics, Saint Petersburg State University, Universitetsky pr. 26, Peterhof, Saint Petersburg 198504, Russia; Smirnov_Michael@mail.ru (M.A.S.); a.toikka@spbu.ru (A.M.T.); 2 Department of Physics, Lappeenranta University of Technology, Skinnarilankatu 34, 53850 Lappeenranta, Finland; Erkki.Lahderanta@lut.fi; 3 Institute of Macromolecular Compounds, Russian Academy of Sciences, Bolshoy pr. 31, Saint Petersburg 199004, Russia; alexander.n.bugrov@gmail.com (A.N.B.); valsvet@hq.macro.ru (V.M.S.); 4 Department of Physical Chemistry, Saint Petersburg Electrotechnical University “LETI”, ul. Professora Popova 5, Saint Petersburg 197376, Russian; 5 Faculty of Physics and Engineering, ITMO University, Kronverskiy pr. 49, Saint Petersburg 197101, Russia; smirnav_2@mail.ru

**Keywords:** segmented block copolymers, microphase separation, atomic force microscopy, small-angle X-ray diffraction, structure–property relationships, membranes, pervaporation

## Abstract

Segmented poly(urethane-imide)s (PUIs) were synthesized by polyaddition reaction and applied for preparation of membranes. Tolylene-2,4-diisocyanate, pyromellitic dianhydride, and *m*-phenylenediamine for chain extension were used to form hard aromatic blocks. Polycaprolactone diols with molecular weights equal to 530 and 2000 g mol^−1^ were chosen as soft segments. The effect of the length of soft segments on the structure, morphology, and transport properties of segmented poly(urethane-imide) membranes were studied using atomic force microscopy, small-angle and wide-angle X-ray scattering, and pervaporation experiments. It was found that a copolymer with a shorter soft segment (530 g mol^−1^) consists of soft domains in a hard matrix, while the introduction of polycaprolactone blocks with higher molecular weight (2000 g mol^−1^) leads to the formation of hard domains in a soft matrix. Additionally, the introduction of hard segments prevents crystallization of polycaprolactone. Transport properties of membranes based on segmented PUIs containing soft segments of different length were tested for pervaporation of a model mixture of propanol/water with 20 wt % H_2_O content. It was found that a membrane based on segmented PUIs containing longer soft segments demonstrates higher flux (8.8 kg μm m^−2^ h^−1^) and selectivity (179) toward water in comparison with results for pure polycaprolactone reported in literature. The membrane based on segmented PUIs with 530 g mol^−1^ soft segment has a lower flux (5.1 kg μm m^−2^ h^−1^) and higher selectivity (437).

## 1. Introduction

Due to desirable mechanical properties, high thermal stability [1,2], chemical resistivity, and low dielectric constant the aromatic polyimides—PI (the class of polyheteroarylenes)—are attractive candidates for application in such fields as fiber optics materials [3,4], microelectronics packaging materials [5], and membranes separation of industrially important gas [6,7] and liquid [8,9] mixtures. However, the dense structure and chemical resistivity lead to a lack of biodegradation, poor solubility and low permeability, which restricts the possible fields of PI application. In this sense, the synthesis of PI modified with extended soft aliphatic blocks, for example, polyesters, has recently attracted attention [10,11]. Among the polyesters, polycaprolactone (PCL) has been well-known since the 1930s as a biocompatible polymer that could be degraded with microorganisms in 2–4 years depending on molecular weight [12,13]. PI and PCL are not compatible and their polymer blends tend to have phase separation. To increase the homogeneity of materials, the segmented block copolymers of PI and PCL were recently synthesized [14]. However, the thermodynamic incompatibility of PI and PCL blocks in such systems is also preserved and promotes the formation of a domain structure [15].

The new segmented polyurethanes and polyimides comprising alternating hard aromatic and soft aliphatic blocks can be proposed as novel and promising types of membrane materials for gas separation and pervaporation. It is known that PUs, dependent on their chemical composition, demonstrate high gas separation properties [16] or high water steam permeability [17]. The segmented structure of macromolecules beneficially influences the transport and mechanical properties of polymer membranes, as was shown for multi-block copolymers [7,18]. It was demonstrated recently that the combination of urea and imide groups in hard blocks increases the mechanical properties of membranes due to physical cross-linking [18]. At the same time, growth of soft-segment content results in the increased permeability of membranes toward CO_2_ [18]. In this sense, developing and verifying methods for investigation of supramolecular organization of segmented polymers is an important task in understanding the relationship between transport properties and the structure of such materials.

Small-angle X-ray scattering (SAXS) is widely applied for studying the microdomain structure of segmented block copolymers [19,20,21,22]. The application with respect to segmented poly(ester-urethane)s of an ellipsoidal domain model for analysis of SAXS data was reported recently [23]. It seems to be an appropriate tool for describing the segmented block copolymer structure. A detailed SAXS analysis by this approach revealed that a hard-segment domain consists of the fringed micelle-like structure. It was demonstrated that the domains increase in the direction of the long axis much more than the short ones during annealing [23].

Atomic force microscopy (AFM) is a versatile method for the investigation of surface topography [24,25], mechanical [26,27,28], and electrical properties [29,30] of different materials on a nanoscale. This method allows for characterization of the phase separation [31,32] and interfacial processes [33] in polymer materials with high spatial resolution. In the early report [34], the tapping-mode AFM was demonstrated to be suitable for visualization of nanometer size domains in the films cast from solutions of segmented polyurethane. The contrast of the AFM image is connected with stiffness differences between hard and soft segments. It was demonstrated that increasing the hard segment length resulted in a better contrast in the AFM image due to higher microphase separation [35]. The bimodal distribution of cantilever adhesion to film surface and visualization of phase structure was demonstrated for a mixed blend of poly(*n*-butyl methacrylate) and polystyrene [36]. As a result, the high potential of AFM in the investigation of microphase separation is often used for support and verification of the SAXS data mentioned above.

In the present work, the combination of AFM and SAXS was used for investigation of novel segmented poly(urethane-imide) (PUI) membranes. In the copolymer, the hard blocks were prepared from tolylene-2,4-diisocyanate, pyromellitic dianhydride, and *m*-phenylenediamine for chain extension and polycaprolactone diols with different molecular weights (530 and 2000 g mol^−1^) were used as the soft blocks. Structural data were used to analyze the transport properties of membranes for separation of the model mixture—*n*-propanol/water. 

## 2. Materials and Methods 

### 2.1. Materials and Preparation of Segmented PUIs

Tolylene-2,4-diisocyanate (95%, *m*_p_ = 20–22 °C, Aldrich, St. Louis, MO, USA, CAS 584-84-9), pyromellitic dianhydride (97%, *m*_p_ = 283–286 °C, Aldrich, CAS 89-32-7), and m-phenylenediamine (flakes, 99%, *m*_p_ = 64–66 °C, Aldrich, CAS 108-45-2) for chain extension were used in the synthesis of segmented poly(urethane-imide)s (PUIs) to form hard aromatic blocks. Polycaprolactone diols (Aldrich, CAS 36890-68-3) with molecular weights (*M*_n_) of 530 and 2000 g mol^−^^1^ were chosen as soft segments. 

**Figure 1:**
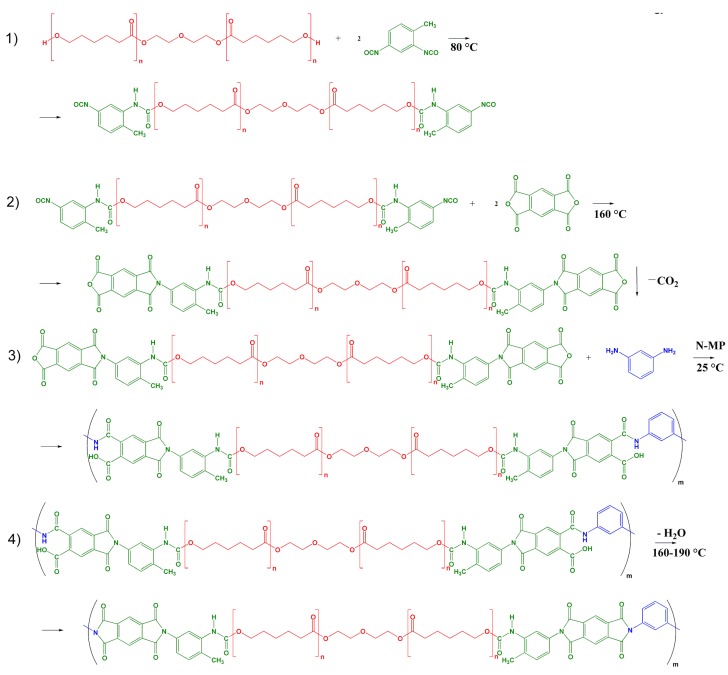
Scheme of the synthesis of segmented poly(urethane-imide)s (PUIs).

**Figure 2:**
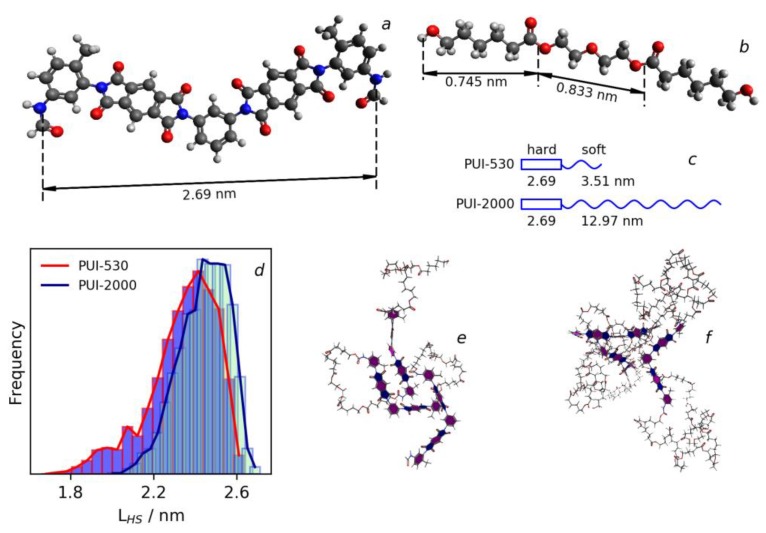
Chemical structure of the repeating unit of the hard segment (**a**) and soft segment (**b**); length of hard and soft segments of block copolymers (**c**); distribution of end-to-end distance of hard segment for macromolecules in solution at 30 °C (**d**); macromolecules of PUI-530 (**e**) and PUI-2000 (**f**), containing three repeating hard-soft blocks after a molecular dynamics simulation in solution.

Synthesis of segmented PUIs was carried out on the basis of the procedure reported in [37], which was modified and included four stages, see Figure 1. First, the end-group macrodiols were terminated by diisocyanate, see Figure 1, reaction 1. For this purpose, 0.75 g (0.0014 mol) of polycaprolactone diols (PCLD) with *M*_n_ = 530 g mol^−^^1^ and 0.49 g (0.0028 mol) of tolylene-2,4-diisocyanate (2,4-TDI) were placed in a thermostated three-necked flask equipped with a mechanical stirrer and argon inlet tube. In the case of the synthesis of segmented PUIs with a longer soft aliphatic block, 1.38 g (0.0007 mol) of PCLD with *M*_n_ = 2000 g mol^−^^1^ and 0.24 g (0.0014 mol) of 2,4-TDI were used. The reaction mixtures were heated to 80 °C and held for one hour with continuous stirring under an argon flow. In the second stage, 0.61 g (0.0028 mol) and 0.3 g (0.0014 mol) of pyromellitic dianhydride were added to macromonomers based on PCLD with *M*_n_ = 530 and 2000 g mol^−^^1^, respectively, terminated with 2,4-TDI, see Figure 1, reaction 2. The resulting mixtures were melted in an inert atmosphere at 160 °C for 2 h and diluted by 10 mL of 1-methyl-2-pyrrolidinone (ACS reagent, ≥99.0%, Aldrich, CAS 872-50-4) during cooling. M-phenylenediamine (m-PDA, 0.15 g (0.0014 mol)) dissolved in 5 mL of 1-methyl-2-pyrrolidinone (N-MP) was added after cooling the flask containing prepolymer based on PCLD with *M*_n_ = 530 g mol^−^^1^ to room temperature, see Figure 1, reaction 3. For the prepolymer with a soft segment of 2000 g mol^−^^1^, the weight of m-PDA dissolved in the amide solvent was 0.07 g (0.0007 mol). The flasks with formed solutions of the copolyesteramic acids were held in a refrigerator overnight and were then supplied with an argon inlet tube, a mechanical stirrer, and a Dean-Stark apparatus with a reflux condenser. Toluene (5 mL) was added to each flask in order to remove the water that is released during imidization in the form of an azeotropic mixture with toluene. The azeotropic distillation was continued for 2 h at 160 °C, 1 h at 180 °C, and 0.5 h at 190 °C, see Figure 1, reaction 4. The polymer solutions were cooled to room temperature, filtered through a Schott filter and degassed under vacuum. Polymeric membranes were formed on glass substrates using a solution casting method. The films were dried at 80 °C for 24 h and then heated stepwise at 140, 160 and 180 °C for 2 h. The thickness of films was 50 μm. The obtained membranes will be denoted as PUI-530 and PUI-2000 in further discussion. The chemical structure of the repeating unit of the hard and soft segments of prepared polymers and their contour length are shown in Figure 2a–c. The contour lengths of hard segment, see Figure 2a, and units of soft segment, see Figure 2b, were measured using Materials Studio 6.0 Package (Accelrys, San Diego, CA, USA) standard tools after optimization of the geometry of segment and units. The contour length of soft segments, see Figure 2c, were calculated on the base of *M*_n_ values for each soft segment and the lengths of the caprolactone and ethylenediol units. 

### 2.2. Characterization Methods

#### 2.2.1. FTIR and NMR Spectroscopy Investigations

In this work the IR Fourier spectrometer Vertex 70 (Bruker, Bremen, Germany) and the ATR reflector (Pike Technologies, Fitchburg, WI, USA) were used. Zn-Se crystals in the form of prisms with an incidence angle of the radiation on the object θ = 45° were used as ATR elements. 

The ^1^H and ^13^C NMR spectra of the segmented PUIs was recorded by a 500 MHz Bruker AVANCE II NMR spectrometer (Bruker, Fällanden, Switzerland) using deuterated DMSO-*d*_6_ as a solvent at 25 °C. 

#### 2.2.2. Microscopic Investigation

The atomic force microscope BRUKER Multimode 8 (Bruker, Santa Barbara, CA, USA) operating in PeakForce TUNA^TM^ tapping mode was used. Scanning was done in PeakForce mode with feedback adjusted automatically by ScanAsyst program protocol. PeakForce parameters such as amplitude and frequency were 150 nm and 2 kHz, respectively. ScanAsyst-Air probe (Bruker, Santa Barbara, CA, USA) with tip radius 2 nm and spring constant 0.47 N m^−1^ was used for topography measurements with setpoint force 2 nN.

#### 2.2.3. Small-Angle and Wide-Angle X-ray Diffraction Study

Small-angle X-ray scattering (SAXS) and wide-angle X-ray diffraction (WAXD) experiments were performed with “SAXSessmc²” (Anton Paar, Graz, Austria) and Rigaku SmartLab 3 (Rigaku Corporation, Tokyo, Japan) diffractometers, respectively. 

#### 2.2.4. Pervaporation Experiments

**Figure 3:**
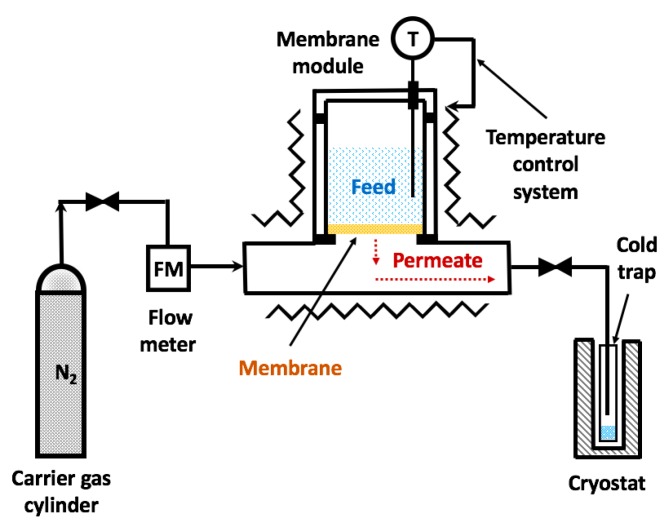
Scheme of the pervaporation set-up.

A laboratory pervaporation system, see Figure 3, was used to study the transport properties of PUIs. The influence of the structure on the membrane transport properties was investigated for the case of pervaporation of model *n*-propanol/water mixture containing 20 wt % of water in feed. The effective membrane area was 1.3 cm^2^. The temperature of the membrane module was 50 °C. The permeate was blown from the membrane surface with dried nitrogen at a flow rate of 60 mL min^−1^ and collected in a glass trap, which was cooled down to −20 °C with a cryostat. Overall flux was determined gravimetrically, while the composition of the permeate was analyzed with gas chromatograph “Chromatec Crystal 5000.2” (Chromatec Company, Yoshkar-Ola, Russia) using a thermal conductivity detector (TCD) and packed column Porapak R (1 m × 3 mm i.d.). The carrier gas was helium with a flow rate of 30 mL/min. Operating temperatures of the column were changed during analysis from 160 until to 210 °C at a rate of 10 °C min^−1^. Temperatures of the vaporizing injector and TCD were 230 and 240 °C, respectively. The fluxes, *J* (kg μm m^−2^·h^−1^), were determined as the amount of liquid transported through a unit of the membrane area per hour normalized by the membrane thickness. Selectivity (*α*) of the membranes respective to water was calculated as *α* = (*x*_p_·*y*_f_)/(*x*_f_·*y*_p_), where *x*_f_, *y*_f_, *x*_p_, and *y*_p_ are the mass fractions of water (*x*) and propanol (*y*) in feed (index *f*) and permeate (index *p*) mixtures, respectively.

#### 2.2.5. Simulation Details

The simulation was performed with Material Studio 6.0 Package (Accelrys). An amorphous cell module was used to prepare the cell containing one macromolecule with three repeating hard-soft blocks in the N-MP solution (15 wt % of polymer). Cells were annealed at 227 °C, 1 atm for 500 ps, then cooled to 30 °C and equilibrated for 400 ps. The density of obtained cells was 1.05 g cm^−3^, which is equal to the experimental density of the same concentration polymer solutions in N-MP, measured gravimetrically. A COMPASS II force field was used. The ensemble was NPT (constant-temperature, constant-pressure) with Nose thermostat and Berendsen barostat. Molecular dynamics simulations were performed at 30 °C for 5 ns and results were used for estimation of end-to-end distance of hard-segments. The result conformation of multi-block copolymer chains after 5 ns simulation and distributions of end-to-end distance of hard segments during 5 ns Molecular Dynamics simulation are shown in Figure 2d.

## 3. Results and Discussion

### 3.1. FTIR and NMR Spectroscopy of Segmented PUIs

**Figure 4:**
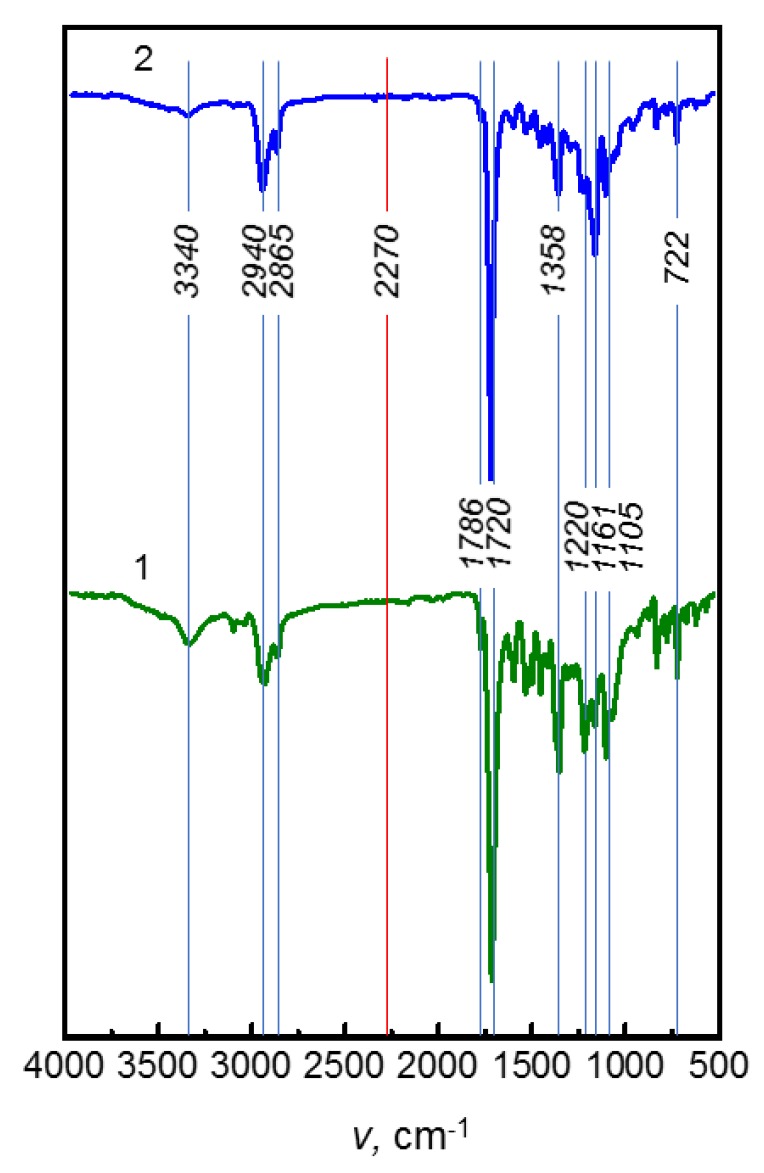
FTIR spectra of PUI-530 (**1**) and PUI-2000 (**2**).

The chemical structure of the obtained multiblock copolymers was identified by FTIR spectroscopy, see Figure 4. The peaks near 3340 cm^−1^ (urethane N–H stretching vibrations), 1720 cm^−1^ (asymmetric stretching vibrations of C=O), and 1105 cm^−1^ (urethane C–O–C stretching vibrations) show the formation of the urethane linkage. The absence of the peak at 2270 cm^−1^ in the IR spectra indicates that the NCO groups of macrodiols terminated by 2,4-TDI completely reacted with pyromellitic dianhydride (PMDA) [38]. The existence of aromatic imide rings in the polymer backbone due to imidization is confirmed by the absorption at 1786 cm^−1^ (symmetric stretching vibrations of C=O), 1720, 1358 cm^−1^ (symmetric stretching vibrations of C–N–C), and 722 cm^−1^ (ring deformation) [39]. The peaks observed at 2940 and 2865 cm^−1^ were assigned to asymmetric and symmetric stretching vibrations of the aliphatic CH_2_ group; 1220 cm^−1^ to coupled C–N and C–O stretching vibrations of the urethane group and 1161 cm^−1^ to C–O–C stretching vibrations in ester [40].

**Figure 5:**
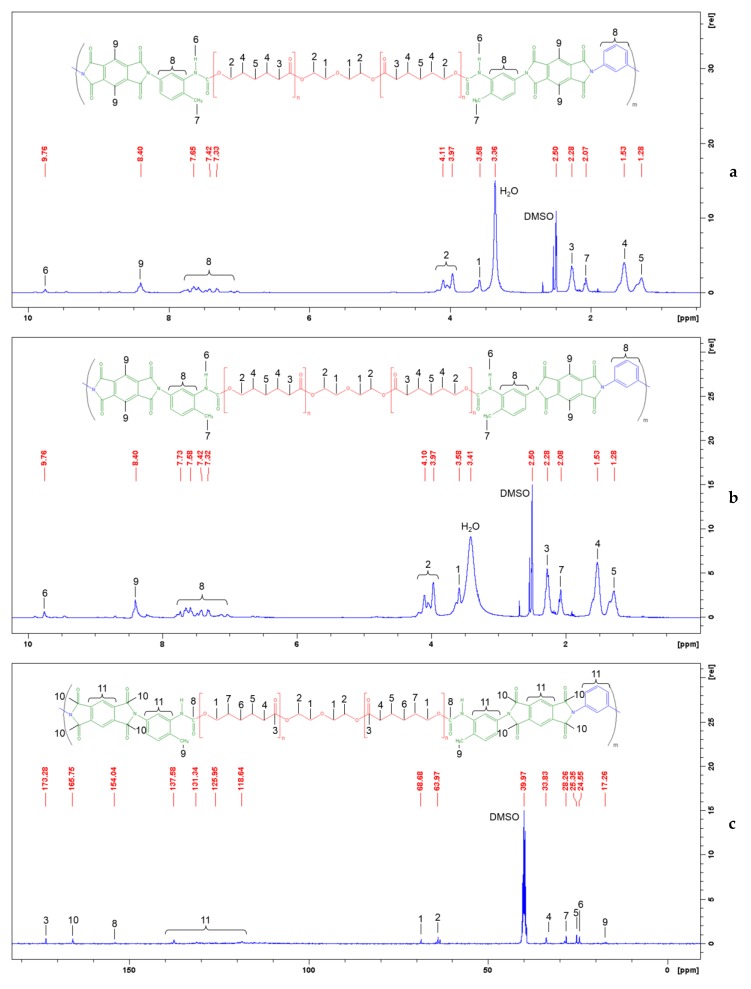

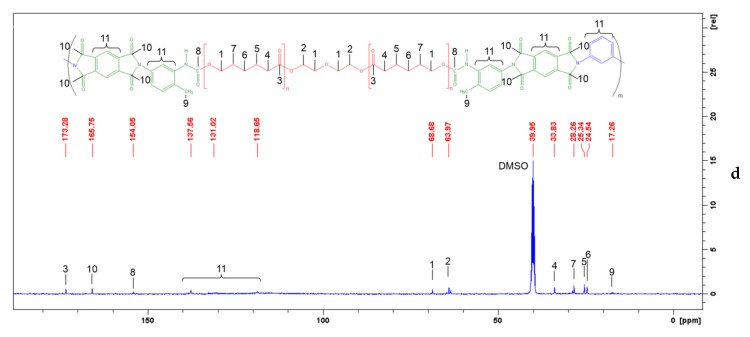
^1^H (**a**,**b**) and ^13^C (**c**,**d**) NMR spectra of the segmented PUIs based on polycaprolactone (PCL) with a molecular weight of 530 (**a**,**c**) and 2000 (**b**,**d**).

The ^1^H and ^13^C NMR spectra of the obtained PUIs are shown in Figure 5. The singlet at 9.76 ppm in ^1^H NMR spectra, see Figure 5a,b, corresponds to NH protons of the urethane group [41]. The two aromatic protons of the pyromellitimide fragment exhibited a signal at 8.40 ppm while the multiplets in the range 7.04–7.81 ppm could be assigned to the protons in the benzene ring of 2,4-TDI and *m*-PDA [42]. Aliphatic CH_3_ protons of diisocyanate in the PUI backbone are observed at 2.07 ppm. The CH_2_ proton signals of PCL are around 4.11–3.97, 3.58, 2.28, 1.53, and 1.28 ppm [43], as shown in Figure 5a,b.

In the ^13^C NMR spectra, the doublet at 154.05 ppm corresponded to the urethane carbonyl groups, as shown in Figure 5c,d. The second doublet was observed at a much higher chemical shift (165.75 ppm) as expected for the imide C=O groups [44]. Carbons in the benzene rings of PMDA, 2,4-TDI and *m*-PDA have signals located between 118.65 and 137.56 ppm. The methyl carbon attached to the aromatic ring in the 2,4-TDI structure is present in the spectra at 17.26 ppm [45]. The signals of typical carbonyls of the PCL ester group were clearly observed at 173.28 ppm. Chemicals shifts at 63.97 and 69.1 ppm correspond to the C2 and C1 carbons in the diethylene glycol moiety of PCL. The signals at 33.83, 28.26, 25.34, and 24.54 ppm correspond to the methylene carbons of PCL [46].

### 3.2. Investigation of Morphology with AFM

**Figure 6:**
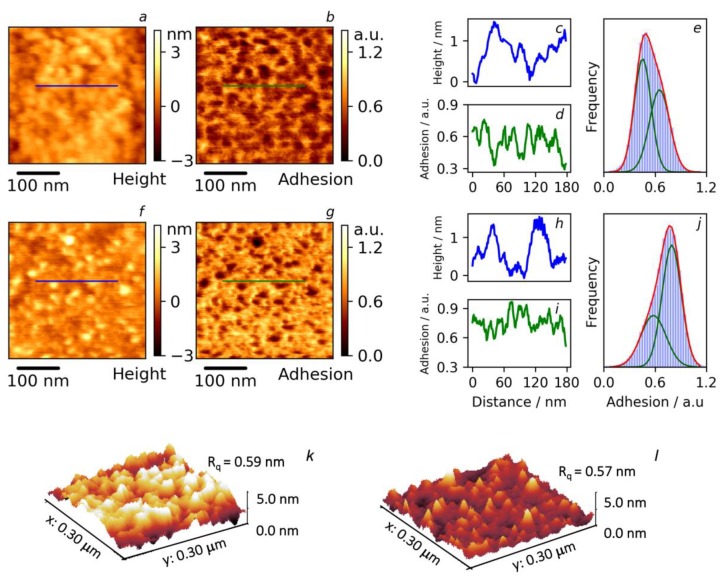
AFM results for PUI-530 (**a**–**e**,**k**) and PUI-2000 (**f**–**j**,**l**): surface topography (**a**,**f**), adhesion maps (**b**,**g**), topography profiles (**c**,**h**), adhesion profiles (**d**,**i)**, distributions of adhesion values (**e**,**j**) and 3D images (k,**l**).

The topography maps demonstrate the smooth surface with the range of heights 4–6 nm for both samples with no significant difference between PUI-530 and PUI-2000, see Figure 6a,c,f,h. The stickier regions in the less sticky matrix and the less sticky regions in the stickier matrix are seen on the adhesion map of PUI-530, shown in Figure 6b, and PUI-2000, shown in Figure 6g, respectively. Assuming the stickier regions are attributed to the soft-segment phase, the difference between images can be connected with the formation of soft domains in the hard-segment based phase for PUI-530 and hard domains in the soft-segment phase for PUI-2000. The difference in the dispersed phase is explained by the composition of segmented copolymers: 51 wt % of hard segments in PUI-530 and only 22% for PUI-2000. The phase separation results in the bi-modal character of the distribution of adhesion values, see Figure 6e,j. The bi-modal character is confirmed by a good fitting of distributions with two Gaussian peaks, which correspond to the adhesion of soft and hard segment phases, see Figure 6e,j. The opposite asymmetry of adhesion distributions for PUI-530 and PUI-2000 is connected with a higher impact of hard-segment phase and soft-segment phase, respectively (see green lines in Figure 6e,j). The smaller spatial periodicity of hard- and soft-domain phases of adhesion maps for PUI-2000 is seen in profiles in Figure 6d,i, which agrees with the results of SAXS investigations that will be discussed further (see Section 3.3). 3D AFM images of prepared membranes are given in Figure 6k,l for PUI-530 and PUI-2000, respectively. The relief scale for both samples is in the range 4–6 nm, which leads the low roughness of surface: *R*_q_ = 0.59 and 0.57 nm for PUI-530 and PUI-2000, respectively. At the same time, the difference can be noticed in the 3D images. For PUI-530 the hollows with sizes 10–20 nm and nodules connected to each other with sizes 20–40 nm can be observed. In contrast to PUI-530, for PUI-2000 the smaller and stand-alone nodules (8–24 nm) are seen, as shown in Figure 6l. It is reasonable to assume that nodules are formed from hard domains during the drying of the film. In this case, it can be concluded that the surface morphology of the segmented PUIs represents the distribution of soft and hard domains in the samples: soft domains in the hard domain matrix for PUI-530 and hard domains in the soft domain matrix for PUI-2000. This conclusion agrees with other results.

### 3.3. SAXS and WAXD Investigations

**Figure 7:**
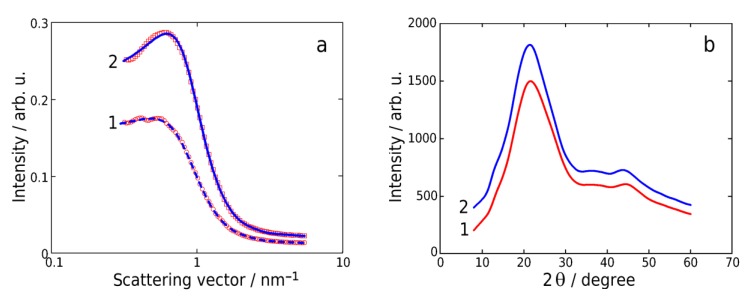
Small-angle (**a**) and wide-angle (**b**) scattering patterns of PUI-530 (1) and PUI-2000 (2) samples.

SAXS curves measured in the range of scattering vector *q* = 0.3–7 nm^−1^ demonstrate a shoulder and the peak for PUI-530 and PUI-2000, respectively, see Figure 7a. Here, the scattering vector *q* is calculated as 4π(sinθ)/λ, where 2θ is the scattering angle and λ is the X-ray wavelength. Scattering occurs upon the changing of electron density on the boundary between soft- and hard-segment domains. The model suggested for segmented block copolymers in the work [23] was used for the fitting of experimental SAXS results in order to evaluate the parameters of the supramolecular organization of segmented PUIs. According to this model, the internal structure is represented as a frozen dispersion of ellipsoidal domains in the polymer bulk. The domains and the surrounding media have a different electron density which results in the scattering of X-rays on the boundary between these two phases. Domains are randomly oriented in the bulk of the sample and have a shape of ellipsoid of revolution. Along with scattering on domains, the model takes into account the scattering on the fluctuation of electron density with correlation radius ξ [47]. It is assumed that there is no interference between scattering from fluctuations and ellipsoid domains. In this case, the intensity of scattering is represented as:

(1) $$I\left(q\right)=A_{1}I_{ell}\left(q,a,\nu \right)S\left(q,R_{HS},\varphi \right)+A_{2}{\left[1+{\left(q\xi \right)}^{2}\right]}^{-2},$$

where *A*_1_ is the coefficient proportional to the contrast of electron density on the domains/continuous phase boundaries and concentration of domains, *A*_2_ is the coefficient representing the contrast of electron density for fluctuations with correlation radius ξ and concentration of fluctuations. The structural factor *S*(*q*,*R_HS_*,φ) takes into account the interference contributions to the diffraction from the spatial distribution of domains, where *R_HS_* is the radius of hard spheres and φ is the volume fraction of hard spheres. The structural factor *S*(*q*,*R_HS_*,φ) in the case of hard spheres is derived on the basis of a Percus-Yevick approximation and has a complicated analytic form (for more details see [48]). The function *I_ell_*(*q*,*a*,ν) is the intensity of scattering (*I*(*q*)) from the uniform ellipsoid of revolution with semiaxes *a*, *a*, ν*a* and unit electron density [49]:

(2) $$I_{ell}\left(q,a,\nu \right)={\left(V_{ell}\right)}^{2}\int_{0}^{1}\Phi^{2}\left[qa\sqrt{1+x^{2}\left(\nu^{2}-1\right)}\right]dx,$$

where $\Phi \left(t\right)=3\left(\sin\left(t\right)-t\cos\left(t\right)\right)/t^{3}$ is the amplitude of scattering from a uniform sphere and $V_{ell}=\frac{4\pi }{3}\nu a^{3}$ is the volume of the ellipsoid. The best fitting was achieved at ν < 1, see Figure 7a, e.g., for the flattened ellipsoids.

The optimal set of parameters obtained with fitting of experimental SAXS data for PUIs samples with the described model are given in Table 1. The geometry of ellipsoid domains (*L*_1_ and *L*_2_ values for the larger and smaller axes respectively) can be found as *L*_1_ = 2*a*, *L*_2_ = 2ν*a*. The average distance *D* between centers of domains is given as:

(3) $$D=\sqrt[{3}]{4\pi R_{HS}^{3}/\left(3\varphi \right)}.$$

The errors given in Table 1 for these parameters were estimated with Monte Carlo simulations. A good agreement between the fitting and experimental data is seen in Figure 7a, where experimental results are shown with red squares and fitted curve with blue lines. The important structural parameters obtained after application of this model are the following: (1) *L*_1_ is the long axis (diameter of a round section) of ellipsoidal domains: 5.47 and 5.12 nm; (2) *L*_2_ is the short axis (diameter perpendicular to the round section) of ellipsoidal domains: 1.83 and 2.01 nm and (3) the average distance between domains centers: 14.8 and 12.2 nm for the samples PUI-530 and PUI-2000, respectively. The short axis is different for the investigated samples, which can be attributed to the different nature of the ellipsoidal domains. Because of the flexibility of the hard segment, see Figure 2e,f, the distance between its ends is less than 2.69 as is shown by the results of the simulation given in Figure 2d. The maximum in the distribution of the end-to-end distance in the hard segment is in the range of 2.4–2.5 nm for both segmented block polymers. Anyhow, this value is still higher than the smaller axis of ellipsoidal domains. At the same time, the domains size obtained from SAXS for PUI-2000 is close to the length of the hard segment. Thus, it can be proposed that the size of the small axis of domains in this sample can be connected to the average distance between ends of the hard segment, while for PUI-530 the sample size of domains is less related to the geometry of the hard segment. This is in agreement with AFM results, from which it was proposed that domains of the PUI-530 sample consist of soft segments. 

The WAXD patterns for both samples were similar and demonstrate a diffuse halo with a maximum at 2θ = 21.5°, see Figure 7b. Two peaks at 2θ = 37.0°, 44.5° and a shoulder at 2θ = 13.0° were also seen, which correspond to the interplanar distances *d* = 2.4, 2.0, and 6.8 Å, respectively. It is worth noting that the typical diffraction peaks for crystalline PCL are intensive and positioned at 2θ = 17.2°, 17.7°, and 19.1°, corresponding to (110), (111), and (200) crystal planes [50], respectively. In the WAXD patterns obtained for PUIs, see Figure 7b, these peaks were not observed. This indicates a poor ordering of soft segments in the membrane. Thus, the combination of the PCL blocks with hard aromatic blocks prevents their crystallization.

### 3.4. Pervaporation Experiments

**Figure 8:**
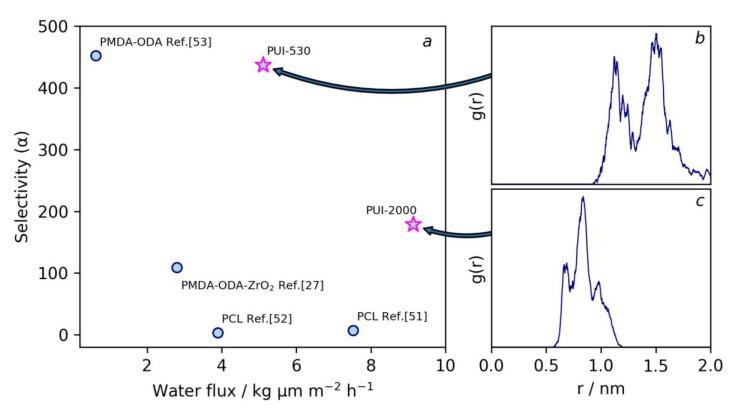
Performance of PUI-530 and PUI-2000 membranes prepared in this work, compared with membranes based on polycaprolactone and pyromellitic dianhydride (PMDA) in the pervaporation dehydration of water/alcohol mixtures (**a**), radial distribution function of distance between C2 carbons of *m*-phenylenediamine rings for PUI-530 (**b**) and PUI-2000 (**c**).

The influence of molecular structure on the transport properties of segmented PUI membranes was investigated with pervaporation of the model *n*-propanol/water mixture containing 20 wt % of water in feed. The membranes were tested during 5 days of continuous pervaporation experiment. It was found that the permeate contains 99.1% and 97.8% of water for PUI-530 and PUI-2000 membranes, which means separation factors of 437 and 179, respectively, were obtained. The fluxes through membranes were 5.1 and 8.8 kg μm m^−2^ h^−1^ for PUI-530 and PUI-2000, respectively. The results presented in this work demonstrate the promising potential of PUI membranes in the separation of a water/alcohol mixture when compared to homopolymer membranes with a comparable chemical composition, which are used for the same purpose, see Figure 8. As can be found in the literature, membranes based on PCL demonstrate high permeability but very low selectivity during dehydration of *i*-propanol [51] or ethanol [52] via pervaporation. This can be connected with the low density of this polymer and hydrophobic nature of the –CH_2_)_5_– parts of its backbone. As opposed to PCL-based films, the polyheteroarylene membranes based on PMDA demonstrate a high selectivity toward water accompanied with rather low fluxes [53]. Some improvement can be achieved by preparation of mixed matrix membranes of polyheteroarylene with inorganic oxide nanoparticles [27], but as can be seen in Figure 8, the introduction of the PCL soft segment leads to the higher fluxes along with the same or higher selectivity than for PMDA-based mixed matrix membranes.

Higher flux can be explained by the lower density of the PUI-2000 polymer in comparison with PUI-530: 1.13 and 1.43 g cm^−3^, respectively. Moreover, as it was found from WAXS measurements, hard blocks prevent the crystallization of PCL. The selectivity of both membranes is higher than the selectivity of PCL-based membranes due to the presence of polar polyheteroarylene fragments, which provide a specific water sorption. The significantly higher selectivity of the PUI-530 membrane can be explained as follows. The content of polyheteroarylene fragments is higher in PUI-530 membrane and they form the continuous spatial phase inside the film. The radial distribution functions between centers of hard segments, which was denoted as the distance between C2 carbon atoms of *m*-phenylenediamine rings, were obtained from molecular dynamics simulation in solution, see Figure 8b,c. It is seen that the average distance between hard segments in PUI-530 segments is higher than in the case of a PUI-2000 macromolecule. This is related to the shorter soft segments in PUI-530 that complicate the proper orientation of hard-segments, which is needed for their interaction via π-π-stacking or hydrogen bonding. It can be proposed, that this results in the less ordered packing of hard-segments and, consequently, in higher accessibility of polar sorption centers in the PUI-530 membrane in comparison with PUI-2000. Thus, both these factors can be possible reasons for the high selectivity of the PUI-530 membrane in comparison to PUI-2000. Thus, tunable features of the structure along with biodegradability of polyester blocks makes poly(urethane-imide) films interesting candidates for investigation with the purpose of developing new membranes for dehydration of water/organic liquids mixtures via pervaporation. 

## 4. Conclusions

Membranes based on segmented poly(urethane-imide)s with polycaprolactone soft segments of different molecular weights (530 and 2000 g mol^−1^) were synthesized by a polyaddition reaction. Investigation with AFM reveals that the copolymer with a shorter soft segment (530 g mol^−1^) consists of soft domains in a hard polymer matrix, while the introduction of PCL blocks with a molecular weight equal to 2000 g mol^−1^ leads to the formation of hard domains in a soft matrix. Results of SAXS measurements were fitted according to the model of ellipsoidal domains. The influence of soft-segment length on the average distance between ellipsoidal domains is in qualitative agreement with AFM measurements. WAXD data indicated that the combination of the PCL blocks with hard aromatic blocks prevents crystallization of soft polyester segments. Transport properties of membranes were studied for pervaporation of a model mixture of *n*-propanol/water with 20 wt % H_2_O content. Combination of hard aromatic and soft aliphatic blocks allows the combination of beneficial properties of corresponding homopolymers: segmented membranes demonstrate the high selectivity of polyheteroarylene parts in combination with a high flux of polycaprolactone membranes. It was found that increasing the PCL segment length results in decreasing the membrane’s density, leading to an increase of flux and decrease of selectivity. However, the lowest obtained selectivity for a membrane with the molecular weight of soft segment 2000 g mol^−1^ was 179, which is significantly higher than the selectivity of polycaprolactone membranes toward water that was reported in the literature [40]. At the same time, the flux was even higher for a segmented block copolymer membrane in comparison with polycaprolactone membranes [40]. The increase of flux for segmented poly(urethane-imide) membranes prepared with a 530 g mol^−1^ soft segment in comparison with polyheteroarylene-based membranes can be explained by the hampering of compact packing of pyromellitic dianhydride fragments due to the short length of the soft segment.

## Figures and Tables

**Table 1:** Fitting parameters of the small-angle X-ray scattering (SAXS) curves and structural parameters of domains.

Parameters	Samples	
	PUI-530	PUI-2000
*A*_1_	2.847(5) × 10^−4^	5.280(6) × 10^−4^
ν	0.3350(6)	0.3926(4)
*a*, nm	2.733(2)	2.562(1)
*R_HS_*, nm	3.595(7)	3.367(3)
φ	6.17(2) × 10^−2^	8.84(1) × 10^−2^
*A*_2_	1.496(1) × 10^−2^	2.533(1) × 10^−2^
ξ, nm	5.31(1) × 10^−2^	5.36(1) × 10^−2^
*D*, nm	14.67(3)	12.18(1)
*L*_1_	5.466(4)	5.124(2)
*L*_2_	1.831(4)	2.012(2)
